# *Aspergillus* Thyroiditis after Allogeneic Hematopoietic Stem Cell Transplantation

**DOI:** 10.1155/2015/537187

**Published:** 2015-11-12

**Authors:** Pinar Ataca, Erden Atilla, Pelin Saracoglu, Gulden Yilmaz, Sinem Civriz Bozdag, Selami Kocak Toprak, Meltem Kurt Yuksel, Koray Ceyhan, Pervin Topcuoglu

**Affiliations:** ^1^Ankara University School of Medicine, Department of Hematology, Ankara, Turkey; ^2^Ankara University School of Medicine, Department of Internal Medicine, Ankara, Turkey; ^3^Ankara University School of Medicine, Department of Infectious Diseases and Clinical Microbiology, Ankara, Turkey; ^4^Ankara University School of Medicine, Department of Pathology, Ankara, Turkey

## Abstract

*Aspergillus* thyroiditis is a rare disorder detected in immunocompromised patients during disseminated infections. Early management is essential to prevent high mortality. A 61-year-old allogeneic stem cell male recipient presented with painful thyroid nodular enlargement. He had low TSH and low free T4 levels. The thyroid ultrasound showed a hypoechoic nodule; biopsy indicated suppurative* Aspergillus* thyroiditis. He was successfully treated by amphotericin B.

## 1. Introduction

The thyroid is generally resistant to infections due to its encapsulated location, high iodine concentration, hydrogen peroxide production, and high levels of blood and lymphoid circulation [1]. Predisposing factors for thyroid infection are previous thyroid disease, local trauma, prior infections, and immunocompromising conditions. Infectious acute thyroiditis is a life-threatening disorder, and urgent recognition and treatment should be considered [2].

Fungal thyroid infection, specifically* Aspergillus* thyroiditis, is a rare entity in immunocompromised patients.* Candida*,* Cryptococcus neoformans*,* Coccidioides immitis,* and* Histoplasma capsulatum* are other agents that have caused fungal thyroiditis in previous case reports. Most patients with fungal thyroiditis have disseminated fungal infection, primarily originating in the lungs [3].

Here, we present a patient with* Aspergillus* thyroiditis after allogeneic hematopoietic stem cell transplantation (allo-HSCT) under immunosuppressive treatment.

## 2. Case Report

**Figure 1:**
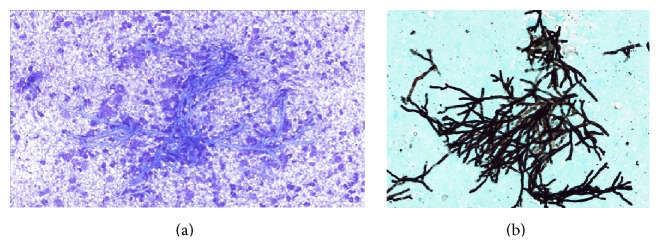
(a) 45-degree angle branching fungal hyphaes within a necroinflammatory background (May Grünwald Giemsa Stain, ×400). (b) Septated and branching fungal hyphaes consistent with aspergillosis (Grocott Methenamine Silver stain, ×400).

A 61-year-old male patient was diagnosed with stage 1 mycosis fungoides in January 2010 and was treated with photochemotherapy, interferon, extracorporeal photopheresis (ECP), and Bexarotene. After 44 months of diagnosis, the patient presented with inguinal lymph node enlargement. The biopsy results revealed large cell transformation. The patient underwent allogeneic stem cell transplantation of peripheral blood from 9/10 HLA-matched unrelated donors with a reduced intensity (fludarabine, cyclophosphamide, ATG, and TBI) conditioning regimen. Posttransplant graft versus host disease (GVHD) prophylaxis was administered in the form of methotrexate (10 mg/m^2^; days 1, 3, 6, and 11) and cyclosporine (3 mg/kg/day). Thirty-two days after transplantation, the patient was admitted to the hospital with acute gastrointestinal (grade 1) and skin GVHD (grade 2). As a result, methylprednisolone 2 mg/kg/day was initiated with a tapering program in addition to cyclosporine and mycophenolate mofetil. Steroid induced diabetes mellitus occurred during follow-up. At 20 months of follow-up, the patient developed chronic extensive GVHD, and, under immunosuppressive therapy and ECP, he presented with fatigue, cough, and fever.* Aspergillus fumigatus* was isolated in sputum culture concomitant with high serum galactomannan antigen levels (1.99 S/CO). CT scan of the thorax revealed 25 × 20 mm nodules in the left medial lower lobe. We replaced the voriconazole treatment with liposomal amphotericin B (5 mg/kg) due to severe hallucinations. However, after one week of treatment, painful thyroid nodular enlargement was detected. The following thyroid function tests were performed: TSH: 0.29 mU/L (0.3–5 mU/L), free T4: 0.3 ng/dL (0.7–2.1 ng/dL), and free T3: 0.2 ng/dL (0.2–6.5 ng/dL) showing sick euthyroid syndrome. The thyroid ultrasound showed a 5.5 × 4 × 3 cm hypoechoic nodule. Fine needle aspiration (FNA) indicated suppurative* Aspergillus* thyroiditis (Figures 1(a) and 1(b)). With amphotericin B (5 mg/kg) treatment, the patient's serum galactomannan antigen levels were decreased to 1.74, 0.45, 0.31, and 0.1. The pulmonary nodules disappeared and the thoracic nodules regressed and the patient was stable after 6 weeks of treatment with normal thyroid function tests.

## 3. Discussion

Fungal thyroiditis is a rare entity among cases of suppurative thyroiditis.* Aspergillus* is the most common cause, followed by* Candida* [4]. Fungal thyroiditis is detected in immunocompromised patients during disseminated* Aspergillus* infections. Disseminated aspergillosis presents with involvement of various organs, such as the brain, skin, thyroid, bone, kidney, liver, eye, or heart. Hematogenous dispersion mechanisms include neutrophil recruitment, activation of cellular immunity, inhibition of host defense, and suppressed T-cell response [5].

The diagnosis of fungal thyroiditis is challenging, and over 50% of patients present with clinical and laboratory symptoms of thyroid dysfunction [4]. Fever, neck pain, thyroid enlargement, dyspnea, and dysphonia are the most common symptoms. Moreover, the thyroid function test can show variability, such as euthyroidism, hypothyroidism, or hyperthyroidism [4]. The sick euthyroid syndrome is characterized by decreased serum free T3 and T4, low serum TSH indicating profoundly altered negative feedback in the pituitary and hypothalamus [6]. Thyroid FNA, biopsy, cytology, and culture are some of the mandatory diagnostic tests. High serum galactomannan antigen levels support the diagnosis of invasive aspergillosis [7]. With a negative predictive value of 95%,* Aspergillus* galactomannan screening by ELISA excludes disseminated aspergillosis [8]. Our patient presented with painful thyroid enlargement, and his laboratory results indicated euthyroid syndrome. Halazun et al. described normalization of thyroid function within 2 weeks following the initiation of antifungal therapy, similar to our patient [9].

According to the literature,* Aspergillus* thyroiditis is predominantly diagnosed in immunocompromised patients diagnosed with, for example, non-Hodgkin's lymphoma, lupus erythematosus, or chronic granulomatous disease [9–11]. The first case report of* Aspergillus* infection involving thyroid gland was described in 1950 by Grekin et al. in autopsy [12]. Hori et al. conducted a postmortem study of 107 invasive aspergillosis patients (55 with extrapulmonary aspergillosis), and only 13 had (12%) fungal thyroiditis [13]. A review by Denning and Stevens suggested that 9–15% of disseminated* Aspergillus* patients have thyroid involvement at autopsy [14]. Solak et al. presented a case of thyrotoxicosis with hypoactive nodules after renal transplantation with fever, cough, and neck pain. The thoracic CT revealed invasive aspergillosis, while thyroid biopsy indicated a diagnosis of* Aspergillus* thyroiditis. The patient was successfully treated with voriconazole [15].* Aspergillus* thyroiditis was also detected in total thyroidectomy material in immunocompetent patients with no comorbidities and admitted with dyspnea, fatigue, and nodular palpable thyroiditis [16]. Our patient was a high risk patient as a survivor of allogeneic stem cell transplantation followed by multiple immunosuppressive treatments in addition to occurrence of diabetes mellitus.

Delay in diagnosis and treatment may contribute to high mortality rates.* Aspergillus* thyroiditis treatment is not distinct from that for invasive aspergillosis. Typically, the first line of treatment is voriconazole, followed by amphotericin B, caspofungin, posaconazole, or micafungin. There are no definitive data regarding combination therapy. In severely ill patients with poor prognostic features, the recommended combination is voriconazole and caspofungin or micafungin [17].

To our knowledge, our case is the first case of successfully treated* Aspergillus* thyroiditis reported after unrelated allogeneic stem cell transplantation. In conclusion, fungal thyroiditis is detected in immunocompromised patients with fever and thyroid enlargement, and prompt management should be considered.
